# Supplementary figures and images for: Investigating neonatal sepsis: anti-Infectives, diagnostics and Guidelines used in Health sysTems across sub-Saharan Africa – The INSIGHTS study
Source: BMJ Paediatr Open. 2026 Jan 23;10(1):e004132. doi: 10.1136/bmjpo-2025-004132 (PMC12853458; doi:10.1136/bmjpo-2025-004132)

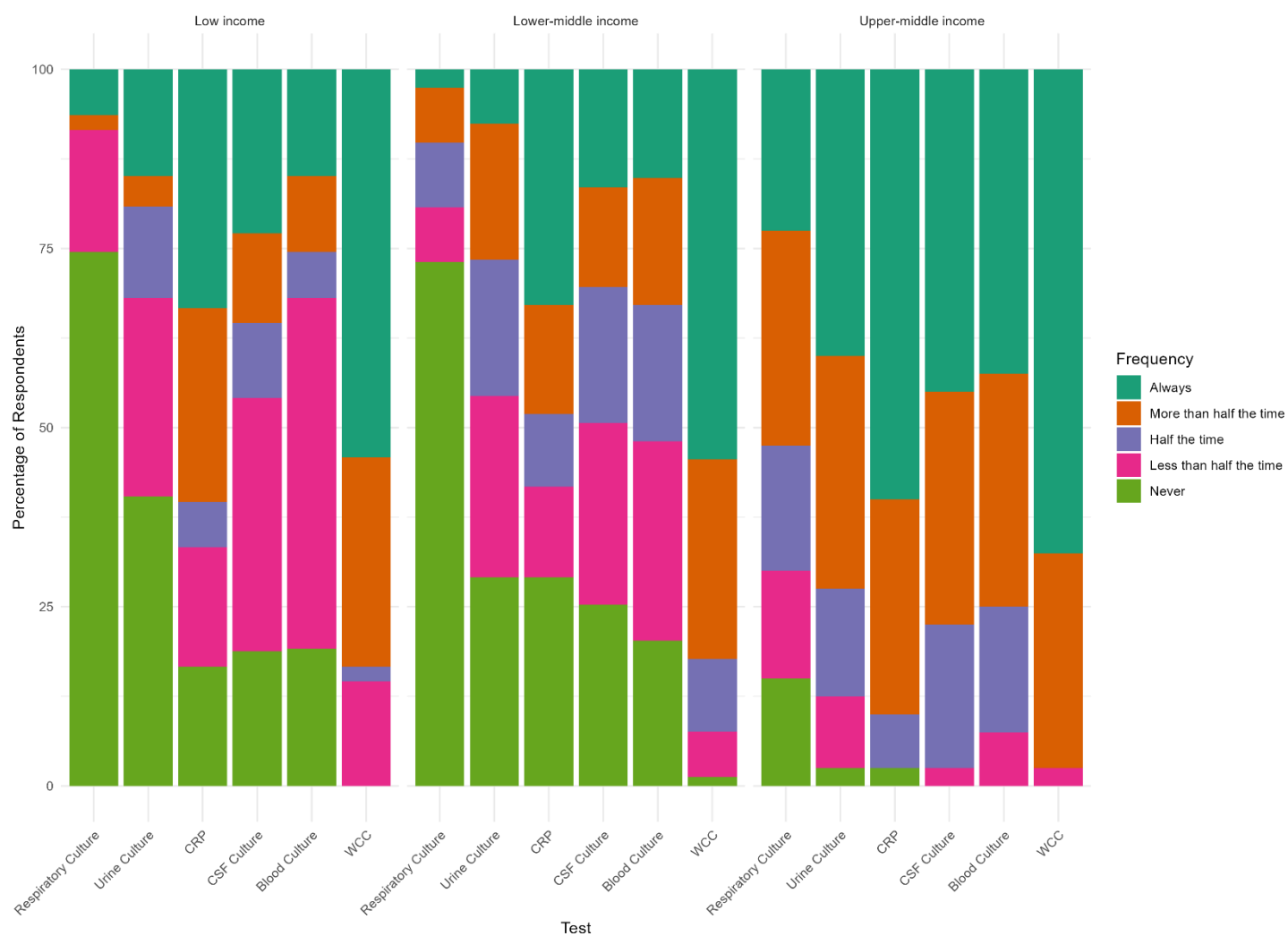

Supplement: online supplemental file 1 [file bmjpo-10-1-s001.pdf]

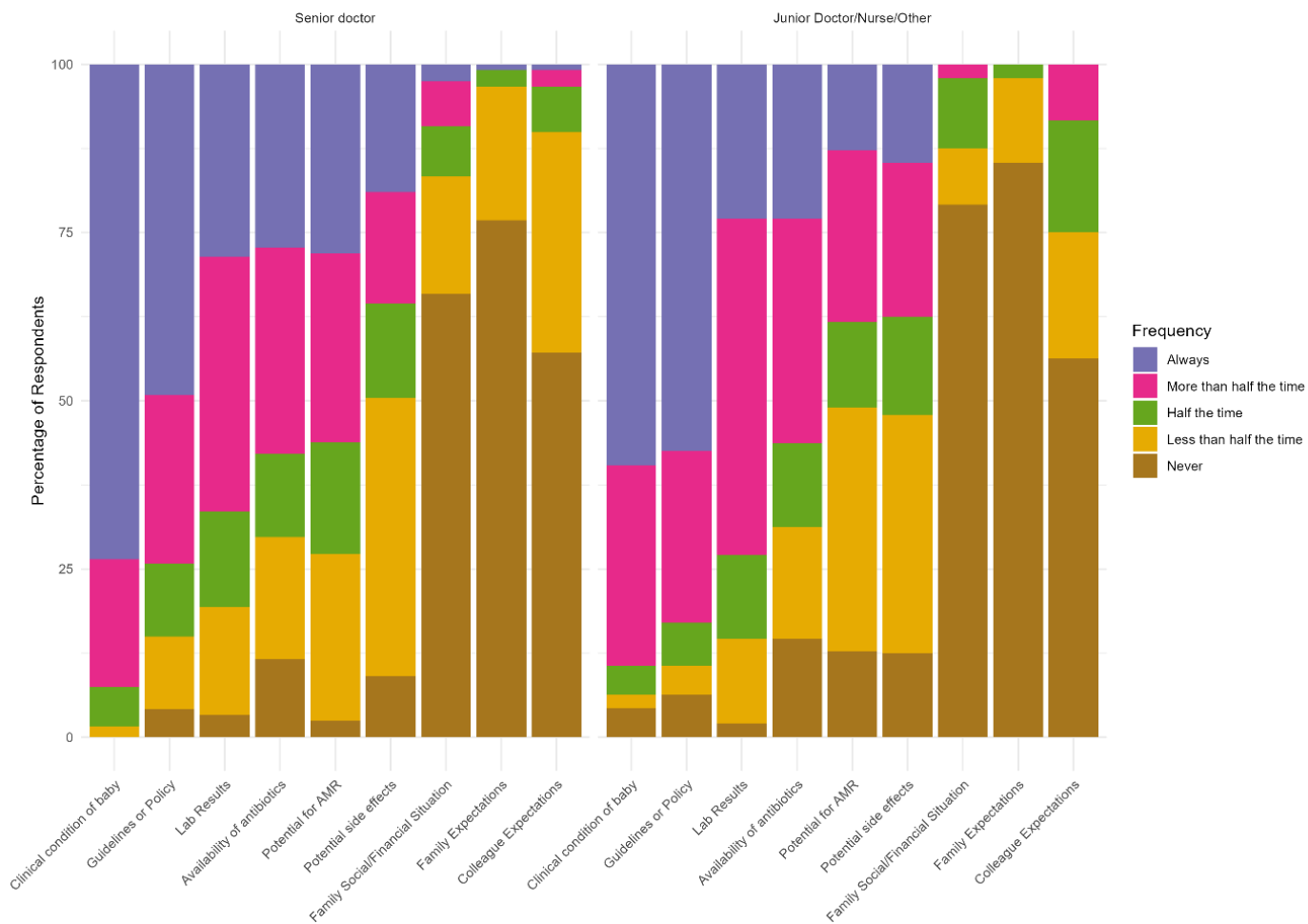

Supplement: online supplemental file 2 [file bmjpo-10-1-s002.pdf]

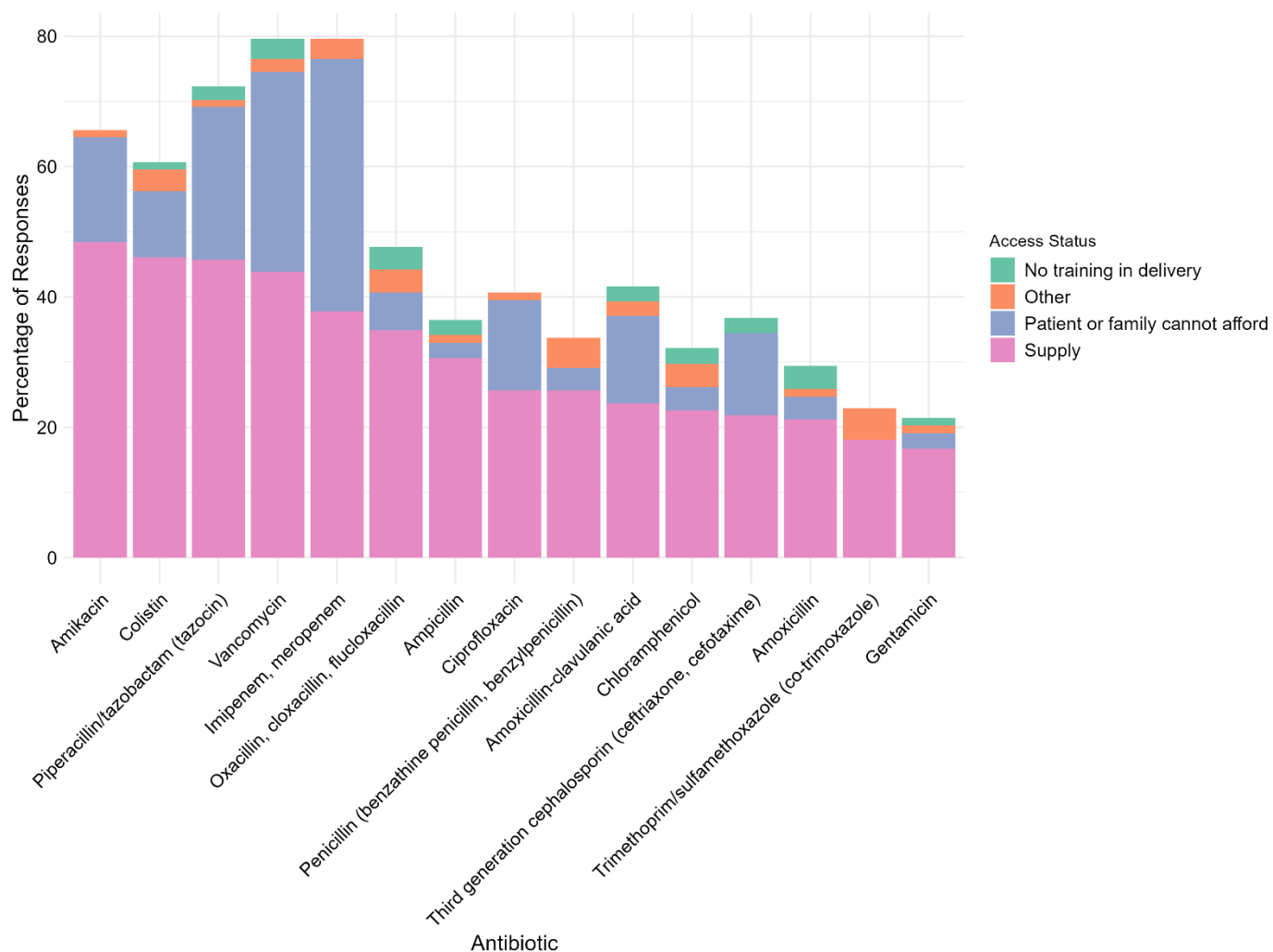

Supplement: online supplemental file 3 [file bmjpo-10-1-s003.pdf]
