# Supplementary material for: Investigating neonatal sepsis: anti-Infectives, diagnostics and Guidelines used in Health sysTems across sub-Saharan Africa – The INSIGHTS study
Source: BMJ Paediatr Open. 2026 Jan 23;10(1):e004132. doi: 10.1136/bmjpo-2025-004132 (PMC12853458; doi:10.1136/bmjpo-2025-004132)
Supplement: online supplemental file 4 [file bmjpo-10-1-s004.pdf]

Exploring practices in diagnostics, antimicrobials and guideline utilisation alongside establishing a minimum surveillance dataset for the management of neonatal sepsis in sub-Saharan Africa

Demographics

1 What is your job role?

- ◯ Senior doctor (Neonatal clinician, paediatrician, consultant)
- ◯ Junior doctor (Intern, medical officer, registrar)
- ◯ Nurse (neonatal/paediatric)
- ◯ Other

1a Please specify

____

2 For how many years have you practised professionally, after graduation?

- ◯ < 5 years
- ◯ 5-10 years
- ◯ 11-20 years
- ◯ >20 years

3 Please specify which country you practise in.

____

4 In what type of healthcare facility do you work?

- ◯ Public
- ◯ Private
- ◯ Both public and private
- ◯ Other

4a Please specify

____

5 In which healthcare place do you practice? (tick all which apply)

- ☐ Primary healthcare facility (e.g., hospital or clinic)
- ☐ District level (secondary level) hospital/healthcare facility
- ☐ Central (tertiary or quaternary) hospital/healthcare facility
- ☐ Laboratory
- ☐ Academic/Research institution (including staff involved in clinical duties)
- ☐ Other

5a Please specify

____

6 What is the average number of neonatal inpatients at your hospital/healthcare facility at any given time?

- ◯ < 20
- ◯ 21-40
- ◯ 41-60
- ◯ 61-100
- ◯ >100

7 How often do you manage patients with neonatal sepsis?

- ◯ Daily
- ◯ Weekly
- ◯ Monthly
- ◯ Yearly
- ◯ Less than yearly

Diagnostics in neonatal sepsis

8. Do you have access to the below diagnostic services to support you in the care of patients with neonatal sepsis?

	Never	Less than half of the time	Half of the time	More than half of the time	Always
Blood white cell count	◯	◯	◯	◯	◯
C-reactive protein	◯	◯	◯	◯	◯
Blood culture	◯	◯	◯	◯	◯
Urine culture	◯	◯	◯	◯	◯
Respiratory (bacterial) culture	◯	◯	◯	◯	◯
Cerebrospinal fluid (CSF) culture	◯	◯	◯	◯	◯

9. How often in the last month have you ordered/performed each test?

	Never	1-5	6-10	11-20	21-50	51-100	>100
Blood white cell count	○	○	○	○	○	○	○
C-reactive protein	○	○	○	○	○	○	○
Blood culture	○	○	○	○	○	○	○
Urine culture	○	○	○	○	○	○	○
Respiratory (bacterial) culture	○	○	○	○	○	○	○
Cerebrospinal fluid (CSF) culture	○	○	○	○	○	○	○

10. How often is the result of this test available to you quickly enough to impact your patients' care?

	Never	Less than half of the time	Half of the time	More than half of the time	Always
Blood white cell count	○	○	○	○	○
C-reactive protein	○	○	○	○	○
Blood culture	○	○	○	○	○
Urine culture	○	○	○	○	○
Respiratory (bacterial) culture	○	○	○	○	○
Cerebrospinal fluid (CSF) culture	○	○	○	○	○

11. How often do you have difficulty with each of the following in terms of microbiology services?

	Never	Less than half of the time	Half of the time	More than half of the time	Always
No laboratory service available	○	○	○	○	○
Lack of consumables to perform tests from the clinical side (e.g. needles, blood culture bottles, lumbar puncture materials)	○	○	○	○	○
Transport/delivery of samples to the laboratory	○	○	○	○	○
Samples not being accepted by the laboratory	○	○	○	○	○
Patients being unable to afford recommended tests	○	○	○	○	○
Tests not being completed by the laboratory	○	○	○	○	○
Laboratory results do not return to me	○	○	○	○	○
Laboratory results received too late to impact patient care	○	○	○	○	○
Laboratory results are not reliable	○	○	○	○	○

Laboratory results are from samples taken after antibiotics have already been administered?	◯	◯	◯	◯	◯
Other	◯	◯	◯	◯	◯

	Never	Less than half of the time	Half of the time	More than half of the time	Always	I don't know about this issue
Lack of consumables to perform tests from the laboratory side (e.g. antibiotic disks, agars, slides)	◯	◯	◯	◯	◯	◯
High workload in the laboratory (sample numbers or staff shortages)	◯	◯	◯	◯	◯	◯

11a If you selected anything other than 'Never' for other, please specify the issue. ______________________________

Antibiotic management in neonatal sepsis

12. Which of these factors influence your decision whether to start antibiotic treatment or not in a neonate?

	Never	Less than half of the time	Half of the time	More than half of the time	Always
Clinical condition of the baby	◯	◯	◯	◯	◯
Expectations of the family	◯	◯	◯	◯	◯
Expectations of colleagues	◯	◯	◯	◯	◯
Social/financial situation of the family	◯	◯	◯	◯	◯
Guidelines/policy	◯	◯	◯	◯	◯
Laboratory results	◯	◯	◯	◯	◯
Potential development of antimicrobial resistance	◯	◯	◯	◯	◯
Availability of any appropriate antibiotics	◯	◯	◯	◯	◯
Potential side effects of antibiotics	◯	◯	◯	◯	◯

13. Once you have made the decision to start antibiotics, what influences your decision on which antibiotic(s) to prescribe in neonatal sepsis?

	Yes	No
Personal experience/training	◯	◯
Advice of neonatal clinicians	◯	◯
Advice of microbiologists	◯	◯

Local guidelines	◯	◯
National guidelines	◯	◯
International guidelines	◯	◯
Clinical condition of the baby	◯	◯
Cost of antibiotics	◯	◯
Availability of antibiotics	◯	◯
Other	◯	◯

13a Please specify

____

14 Do you have any challenges accessing antibiotics in your practice managing neonatal sepsis?

◯ Yes
◯ No

14a How often do you have difficulty accessing each of the following antibiotics to use in patients with neonatal sepsis?

	Never	Less than half of the time	Half of the time	More than half of the time	Always	Would not use this antibiotic
Penicillin (benzathine penicillin, benzylpenicillin)	◯	◯	◯	◯	◯	◯
Ampicillin	◯	◯	◯	◯	◯	◯
Amoxicillin	◯	◯	◯	◯	◯	◯
Oxacillin, cloxacillin, flucloxacillin	◯	◯	◯	◯	◯	◯
Ceftriaxone, cefotaxime (3rd generation cephalosporin)	◯	◯	◯	◯	◯	◯
Gentamicin	◯	◯	◯	◯	◯	◯
Amikacin	◯	◯	◯	◯	◯	◯
Imipenem, meropenem	◯	◯	◯	◯	◯	◯
Ciprofloxacin	◯	◯	◯	◯	◯	◯
Azithromycin, erythromycin	◯	◯	◯	◯	◯	◯
Chloramphenicol	◯	◯	◯	◯	◯	◯
Co-trimoxazole	◯	◯	◯	◯	◯	◯
Vancomycin	◯	◯	◯	◯	◯	◯
Amoxicillin/clavulanic acid (co-amoxiclav)	◯	◯	◯	◯	◯	◯
Piperacillin/tazobactam (tazocin)	◯	◯	◯	◯	◯	◯
Colistin	◯	◯	◯	◯	◯	◯
Fluconazole	◯	◯	◯	◯	◯	◯
Amphotericin B	◯	◯	◯	◯	◯	◯
Aciclovir (acyclovir)	◯	◯	◯	◯	◯	◯

14b What are the difficulties that you have in using these antibiotics?

	Out of stock antibiotic/no supply to healthcare facility	Patient/family unable to afford medications	Inadequate training to deliver the medication	Other	Do not use this antibiotic in practice	No difficulty with access to this antibiotic
Penicillin (benzathine penicillin, benzylpenicillin)	☐	☐	☐	☐	☐	☐
Ampicillin	☐	☐	☐	☐	☐	☐
Amoxicillin	☐	☐	☐	☐	☐	☐
Oxacillin, cloxacillin, flucloxacillin	☐	☐	☐	☐	☐	☐
Ceftriaxone, cefotaxime (3rd generation cephalosporin)	☐	☐	☐	☐	☐	☐
Gentamicin	☐	☐	☐	☐	☐	☐
Amikacin	☐	☐	☐	☐	☐	☐
Imipenem, meropenem	☐	☐	☐	☐	☐	☐
Ciprofloxacin	☐	☐	☐	☐	☐	☐
Azithromycin, erythromycin	☐	☐	☐	☐	☐	☐
Chloramphenicol	☐	☐	☐	☐	☐	☐
Co-trimoxazole	☐	☐	☐	☐	☐	☐
Vancomycin	☐	☐	☐	☐	☐	☐
Amoxicillin/clavulanic acid (co-amoxiclav)	☐	☐	☐	☐	☐	☐
Piperacillin/tazobactam (tazocin)	☐	☐	☐	☐	☐	☐
Colistin	☐	☐	☐	☐	☐	☐
Fluconazole	☐	☐	☐	☐	☐	☐
Amphotericin B	☐	☐	☐	☐	☐	☐
Aciclovir (acyclovir)	☐	☐	☐	☐	☐	☐

14b-iPlease specify

____

15	Do you have pharmacy support available in managing your antibiotic decisions?	◯ Never<br>◯ Less than half of the time<br>◯ Half of the time<br>◯ More than half of the time<br>◯ Always<br>◯ Would not use/consult pharmacy for this
16	Is there an antimicrobial stewardship (AMS) team/action plan specific to your facility?	◯ Yes<br>◯ No<br>◯ Not sure<br>(This would not be in the form of a National Action Plan, it must be facility specific)
17	Are there regular audits or reviews of the use of antibiotics in your facility?	◯ Yes<br>◯ No<br>◯ Not sure

The use of guidelines in neonatal sepsis

18	Do you refer to treatment guidelines in the management of neonatal sepsis?	◯ Yes<br>◯ No
19	Which, if any, antibiotic guideline is used for treating neonatal sepsis at your healthcare facility?	☐ Yes - local guideline (specific to my healthcare facility)<br>☐ Yes - national guideline<br>☐ Yes - WHO guideline<br>☐ Other guideline<br>☐ No guideline
19a	Please specify which guideline	__________________________________
19b	Do you find these antibiotic guidelines applicable to your setting?	◯ Yes<br>◯ No<br>◯ Sometimes
19b-i	Please describe why the antibiotic guidelines are not always applicable to your practice?	__________________________________
19c	Are the antibiotic guidelines used at your healthcare facility regularly reviewed and updated considering new evidence (at least every 2 years)?	◯ Yes<br>◯ No<br>◯ Not sure
20	Have you or your colleagues attempted to author any local or national specific antibiotic guidelines?	◯ Yes<br>◯ No
20a	What challenges or support did you encounter during this process?	__________________________________

20b What factors have influenced this?

- ☐ Not enough time to develop
- ☐ Do not feel these are needed
- ☐ Lack of support from colleagues
- ☐ Lack of support from senior hospital staff e.g. medical superintendent, clinical director
- ☐ Financial constraints e.g. could not afford alternative antibiotics to those currently used
- ☐ Availability of other antibiotic agents/options
- ☐ Lack of data on local antimicrobial resistance patterns to guide local guideline development
- ☐ I do not feel experienced enough to contribute towards local guideline development
- ☐ Other

20b-i Please specify which other factors have influenced this

____

---

21 When treating EARLY ONSET neonatal sepsis at your facility what empiric first line antibiotic regimen:

a. do you use in clinical practice?

____
(Please include agent(s) and route of administration (i.e. IV or oral))

---

21 b. is recommended by your guideline?

____
(Please include agent(s) and route of administration (i.e. IV or oral))

---

22 When treating LATE ONSET HOSPITAL-ACQUIRED neonatal sepsis at your facility what empiric first line antibiotic regimen:

a. do you use in clinical practice?

____
(Please include agent(s) and route of administration (i.e. IV or oral))

---

22 b. is recommended by your guideline

____
(Please include agent(s) and route of administration (i.e. IV or oral))

---

23 When treating LATE ONSET COMMUNITY-ACQUIRED neonatal sepsis at your facility what empiric first line antibiotic regimen:

a. do you use in clinical practice?

____
(Please include agent(s) and route of administration (i.e. IV or oral))

---

23 b. is recommended by your guideline?

____
(Please include agent(s) and route of administration (i.e. IV or oral))

---

24 How often are you able to adhere to the antibiotic guidelines at your facility?

- ◯ Never
- ◯ Very rarely
- ◯ Less than half of the time
- ◯ Half of the time
- ◯ More than half of the time
- ◯ Always

25 If you divert from the antibiotic guidelines, why do you do this?

- ☐ Clinical presentation of the neonate
- ☐ Opinion/concern of colleagues on the condition of the neonate
- ☐ Unavailabilty of the antibiotics suggested in the guideline
- ☐ Aware of antibiotic-resistant organisms not addressed by the guideline
- ☐ Availability of equipment (e.g. cannula) to deliver antibiotic suggested in the guideline
- ☐ No intravenous access for antibiotic delivery
- ☐ Financial considerations for family
- ☐ Never have to divert from antibiotic guidelines
- ☐ Other

25a Please specify

____

26 Do you face any barriers in diverting from antibiotic guidelines if you feel it appropriate to do so?

- ◯ Yes
- ◯ No

26a Describe the issues which you face when diverting from antibiotic guidelines.

____

Surveillance in neonatal sepsis

27. Does your facility collect surveillance data on...?

	Yes	No
Patients with suspected neonatal infection	◯	◯
Patients with confirmed neonatal bloodstream infection or meningitis	◯	◯
Neonatal mortality (crude)	◯	◯
Neonatal mortality from sepsis	◯	◯
Antibiotic use in the neonatal unit	◯	◯
No aspects of neonatal sepsis	◯	◯

27a Who collects surveillance data?

- ☐ Clinicians
- ☐ Hospital records team
- ☐ Pharmacists
- ☐ Public Health
- ☐ Microbiology laboratory
- ☐ National Health Information System
- ☐ Other

27a-i Please specify

____

Comments

28 Do you have any further comments?

____
