# Supplementary material for: Investigating neonatal sepsis: anti-Infectives, diagnostics and Guidelines used in Health sysTems across sub-Saharan Africa – The INSIGHTS study
Source: BMJ Paediatr Open. 2026 Jan 23;10(1):e004132. doi: 10.1136/bmjpo-2025-004132 (PMC12853458; doi:10.1136/bmjpo-2025-004132)
Supplement: Supplementary file 5 [file bmjpo-2025-004132.reviewer_comments.pdf]

PEER REVIEW HISTORY

BMJ Paediatrics Open publishes all reviews undertaken for accepted manuscripts. Reviewers are asked to complete a checklist review form and are provided with free text boxes to elaborate on their assessment. These free text comments are reproduced below.

ARTICLE DETAILS

**TITLE (PROVISIONAL)**	Investigating Neonatal Sepsis: Anti-Infectives, Diagnostics and Guidelines used in Health sysTems across Sub-Saharan Africa - The INSIGHTS Study
**AUTHORS**	Stanley, Jack; Hettle, David; Poffley, Rachel; Bolton, Larisse; Gres, Emelyne; Coelho, Isabel; Ra'ana Hussain, Syeda; Hedible, Gildas; Chimhini, Gwendoline; Leroy, Valeriane; AMORISSANI, FOLQUET; Fitzgerald, Felicity; Dramowski, Angela

VERSION 1 - REVIEW

**REVIEWER NAME**	Berhane, Melkamu
**REVIEWER AFFILIATION**	None disclosure
**REVIEWER CONFLICT OF INTEREST**	No
**AI DISCLOSURE**	No, I have not used AI
**DATE REVIEW RETURNED**	20-Oct-2025

**GENERAL COMMENTS**	Comments/suggestions to the authors<br>General comments: The paper is well written and language is clear. The major issue with the paper is the originality and novelty of the study. The study investigated an already well known and well investigated area, so there is nothing readers could gain by reading this article.<br>Originality: The work tried to investigate what is already well known and is there in the published literature and is just confirming the same issue; it doesn't add anything new to the existing literature.<br>Importance of the work to readers: the work matters to readers specially to policy makers. However, it is an already very well known fact, so, there is nothing new in the findings that could impact clinicians, researchers, academicians or policy makers.<br>Evidence based medicine: the evidence presented are scientific and reliable.<br>Research question: clearly defined and well addressed.<br>Study design: is appropriate.<br>Participants: the methods used to approach the participants is not well described, in fact they have mentioned that they used purposive sampling and snowballing approach but what was the entry point used? Where was the online survey posted (how did people access them)? It was not clear why they didn't proportionally include all the levels of healthcare facility/workers as they mentioned this as the limitation of the study. Plus, participants were not adequately described E.g. How did they define senior Vs junior doctor? What does other refer to with regard to the type of health worker?<br>Methods: Described well despite the above mentioned limitation.<br>Results: presented well and are credible. But the major issue is there is nothing new here and it is in line with the several studies conducted so far.

	Interpretation and conclusion: appropriately done but it is not different from what is there in the existing literature.<br>Abstract/summary/key messages: reflect what are presented in the paper but all well known as mentioned above.<br>Reporting guideline: fine.

**REVIEWER NAME**	Jindal, Atul
**REVIEWER AFFILIATION**	All India Institute of Medical Sciences, Pediatrics
**REVIEWER CONFLICT OF INTEREST**	No
**AI DISCLOSURE**	No, I have not used AI
**DATE REVIEW RETURNED**	14-Nov-2025

**GENERAL COMMENTS**	Summary and Significance<br>This multi-country clinician survey addresses a critical gap in neonatal sepsis management across sub-Saharan Africa (SSA), a region significantly impacted by elevated neonatal mortality rates and increasing antimicrobial resistance (AMR). The study evaluates clinicians' access to diagnostics, antibiotics, and guideline usage, providing actionable insights pertinent to policymakers, healthcare professionals, and researchers.<br>Major Strengths<br>The survey encompasses 40 out of 48 SSA countries, making it one of the most geographically comprehensive assessments to date and presenting a wide-ranging overview of neonatal sepsis care within the region. By examining operational barriers—including diagnostic delays, antibiotic accessibility, and the influence of limited AMR data on guideline development—the study delivers findings that are highly relevant and well positioned to inform both regional policy discussions and global guideline revisions. Detailed descriptions of survey development, piloting, multilingual deployment, and ethical approval are provided. Stratification by country income classification and facility type enhances the analysis.<br>Primary Limitations<br>While professional networks and snowball sampling offer practical benefits, they likely lead to overrepresentation of clinicians from urban or central facilities and exclude non-physician practitioners who often deliver frontline neonatal care. The absence of a survey denominator or response rate impedes assessment of representativeness and potential selection bias.<br>Despite piloting, there is insufficient information regarding content, construct, and translation validation. The study would be strengthened by explicit adherence to reporting standards such as CROSS or STROBE, along with provision of the complete survey instrument as supplementary material.<br>The reporting relies primarily on percentage responses, with minimal quantitative comparison among subgroups and without confidence intervals for key findings. Additional statistical analysis by cadre, facility type, or country income would enhance interpretability and generalizability.<br>The study is confined to reported perceptions and practices, which may not accurately reflect actual clinical behavior or patient outcomes. Social desirability bias is possible, particularly regarding self-reported high guideline adherence. A more thorough discussion of the relationship between guideline dependence and observed discrepancies with local epidemiology and AMR, as well as the risks associated with empiric use of ineffective antibiotics and restricted access to escalation agents, would be beneficial.

	Additional Comments and Recommendations<br>Explicitly address and, if feasible, quantify the involvement of nurses and other cadres, given their crucial role, especially in lower-level health facilities.<br>Include the complete instrument—in all utilized languages—as supplementary material, describe the translation process in detail, and indicate measures taken to ensure cross-cultural validity.<br>Present confidence intervals alongside percentage data. When possible, undertake subgroup analyses and consider confounding factors.<br>Clarify confidentiality and anonymity protocols for online survey respondents in addition to the ethics statement provided.<br>Recommendations: Expand recommendations to encompass practical strategies for improving data systems, sharing local AMR data, strengthening guideline review processes, and addressing workforce and infrastructure deficiencies.<br>Limitations Section: Further elaborate on limitations regarding sample representativeness, generalizability, and potential biases stemming from self-reporting and selection methods.<br>Verdict<br>This manuscript constitutes a significant and original contribution with notable clinical and policy implications. Its overall impact would be further enhanced by increased methodological transparency and strict adherence to established reporting guidelines.

VERSION 1 – AUTHOR RESPONSE

	Reviewers Comments	Authors Response
**1a**	Novelty of the study “there is nothing readers could gain by reading this article”<br><br>Originality “the work tried to investigate what is already known… doesn’t add anything to existing literature<br><br>“Nothing new in the findings that could impact clinicians, researchers, academics or policy makers”	We politely challenge this assertion about our work. While the management of neonatal sepsis is known to be challenging in this context, there is sparse understanding of the specifics contributing to these challenges and in particular, how guidelines are used. As we highlight in our introduction, there are concerns that current guidelines do not reflect the realities of local AMR patterns and how sepsis amidst this landscape must be managed (references 13-16). This survey is the largest in the field with regards to response rate, geographical coverage and depth of content (the previous largest survey was Rosa-Mangeret *et al*. [reference 11]). In addition, we draw attention to the comments of reviewer 2, copied verbatim:<br><br>“This multi-country clinician survey addresses a critical gap in neonatal sepsis management across sub-Saharan Africa (SSA)”,

		"The survey encompasses 40 out of 48 SSA countries, making it one of the most geographically comprehensive assessments to date and presenting a wide-ranging overview of neonatal sepsis care within the region. By examining operational barriers - including diagnostic delays, antibiotic accessibility, and the influence of limited AMR data on guideline development - the study delivers findings that are highly relevant and well positioned to inform both regional policy discussions and global guideline revisions."<br><br>While previous studies have explored aspects of our findings (on a smaller scale), none have specifically explored which guidelines are used across Africa, particularly pertinent to understand given the concerns outlined in the introduction that antibiotics used may not match local AMR patterns, in terms of understanding the importance of guidelines in individual neonatal practitioners' practice.
**1b**	Participants. Access to. Methods used to approach the participants not well described. What was the entry point to purposive sampling and snowballing approach?<br><br>Where was the online survey posted?	The entry point to our purposive sampling approach was via the NeoNet Africa and African Neonatal Association networks, as noted in the manuscript. Further detail on these organisations are provided in the text, but are two key pan-African neonatal organisations, likely to include a large number of potential respondents who would meet our inclusion criteria.<br><br>Alongside this purposive approach, known individual neonatal or paediatric associations in the region were also contacted (45 organisations as mentioned in the original manuscript) to distribute to their members if they felt appropriate. The authors of our paper do not know if each of these organisations have specific email distribution lists, but assume that they have methods to communicate with their members and therefore to distribute our survey as they see appropriate to relevant individuals and colleagues.<br><br>Having distributed the invitation through the email and organisation lists, any recipients were encouraged to forward the survey to colleagues who would also meet the inclusion criteria, thereby enabling a modified snowball dissemination (modified as participants were not directly referred on by a single other participant, instead we utilised the farthest reaching dissemination approach we could offer by approaching the above organisations to disseminate via mailing lists and using this access to relevant individuals, to refer onwards appropriately - known to be an effective method in accessing hard-to-reach populations in research

		(Oldhoff-Nuijsink C, Fransen MP, Peute LW, Derksen ME. Strategies for engaging "hard-to-reach" populations in a panel for digital health research: A qualitative study among experts. PLOS Digit Health. 2025 Oct 9;4(10):e0001033.). Lines 132-44 have been re-ordered and added to, aiming to clarify this for readers' benefit.<br><br>The survey was posted on REDCap, an online server. This has been clarified (lines 157-9). Thank you for highlighting.
**1c**	Participants. Not adequately described. How did they define senior Vs junior doctor? What does other refer to with regard to the type of health worker?	Given that our survey covers so many countries and healthcare settings senior doctors are not likely to be exactly the same in each setting. We provided examples of those who might fulfil each criteria in our survey as follows: Senior doctor (Neonatal clinician, paediatrician, consultant), Junior doctor (intern, medical officer, registrar). We allowed responders to decide ultimately though to allow them to reflect their local context. This is made explicit in the updated manuscript lines 171-4.
**2a**	While professional networks and snowball sampling offer practical benefits, they likely lead to overrepresentation of clinicians from urban or central facilities and exclude non-physician practitioners who often deliver frontline neonatal care.<br><br>The absence of a survey denominator or response rate impedes assessment of representativeness and potential selection bias.	We agree with this critique and acknowledge that it is a potential shortcoming of our work. This point was addressed in the penultimate paragraph of our discussion in the original manuscript. We have added an additional comment to make this more explicit (line 406).<br><br>We cannot provide a response rate without knowing every single person who viewed the material which is impossible due to the fact that we employed snowball sampling. This is a limitation but not one that we or any survey can achieve, without being incredibly restrictive in our sampling method, which would unjustifiably limit the generalisability of our work. We have made the first paragraph of the results section clearer with regards to excluded responses to address excluded responses more explicitly. Given the nature of the survey which prevented incomplete responses the denominator is typically 169 except in instances where follow on questions would stem from an earlier answer. In these instances the denominator is provided. This methodology is made more explicit in the final section of the methods (lines 185-7).

**2b**	Despite piloting, there is insufficient information regarding content, construct, and translation validation.<br><br>The study would be strengthened by explicit adherence to reporting standards such as CROSS or STROBE, along with provision of the complete survey instrument as supplementary materials.<br><br>Linked recommendation:<br><br>Include the complete instrument—in all utilized languages—as supplementary material, describe the translation process in detail, and indicate measures taken to ensure cross-cultural validity.	Thank you for this suggestion. In response we have expanded the Methods section (lines 120-90) covering survey/study design to encompass additional detail on survey development (including consideration of content and construct validity) and translation.<br><br>Thank you for raising the CROSS/STROBE criteria. Given the nature of our survey we have explicitly added details on the elements of CROSS criteria which we had not clearly addressed, which were largely content, construct and translation validity so have been addressed as above, as well as mentioning being guided by the CROSS criteria in the manuscript (lines 189-90).<br><br>We have updated the supplementary material to include the survey instrument in all languages.
**2c**	The reporting relies primarily on percentage responses, with minimal quantitative comparison among subgroups and without confidence intervals for key findings. Additional statistical analysis by cadre, facility type, or country income would enhance interpretability and generalizability.<br><br>Linked recommendation:<br><br>Present confidence intervals alongside percentage data. When possible, undertake subgroup analyses and consider confounding factors.	Thank you for highlighting. We have included confidence intervals for all percentages which provides further numerical context.<br><br>The focus of our work is to present a summary of practice and barriers. Statistical subgroup analyses, whilst interesting, would be testing hypotheses that differences existed between different groups of respondents, which is not the aim of our work. We are seeking to understand how guidelines, specifically the WHO guidelines, are utilised and received across the region of Sub-Saharan Africa.<br><br>We have included two plots as supplementary material which address subgroup analyses by income status of respondent's country of practice and seniority. These are included as supplementary rather than main figures because they are not the primary focus of this piece.
**2d**	The study is confined to reported perceptions and practices, which may not accurately reflect actual clinical behavior or patient outcomes.<br><br>Social desirability bias is possible, particularly regarding self-reported high guideline adherence.	We acknowledge this and have made more explicit reference to desirability and recall bias (lines 395-7)<br><br>See above

	A more thorough discussion of the relationship between guideline dependence and observed discrepancies with local epidemiology and AMR, as well as the risks associated with empiric use of ineffective antibiotics and restricted access to escalation agents, would be beneficial.	Thank you for highlighting that this issue was not explicitly laid out, as we believe it is a critical reason that this study is important. We have added a paragraph in the discussion (lines 330-43) and a comment in lines 361-3 to incorporate this more comprehensively, and pull out explicitly the critical issues with the misalignment of guidelines, access to diagnostics and antibiotics and local AMR patterns.
**2e**	Explicitly address and, if feasible, quantify the involvement of nurses and other cadres, given their crucial role, especially in lower-level health facilities.	We have added the number of nurses explicitly. This is also available in figure 2 (although was not previously explicitly mentioned). We address the challenges of the respondents being largely senior doctors (i.e. not nurses, and not those at district or primary healthcare level facilities) and the impact which this may have on our results as mentioned in other comments here, and in our discussion (lines 400-6).
**2f**	Clarify confidentiality and anonymity protocols for online survey respondents in addition to the ethics statement provided	Thank you for highlighting. This has been expanded lines 162-164.
**2g**	Recommendations: Expand recommendations to encompass practical strategies for improving data systems, sharing local AMR data, strengthening guideline review processes, and addressing workforce and infrastructure deficiencies.	Thank you for this comment. We recognise that there are several recommendations which this could prompt so have added a short section in lines 413-20. However, these possibilities have also been outlined more comprehensively in a recent study (as now referenced in line 415 – Dramowski *et al*. (2025)) so we have added this reference.
**2h**	Limitations Section: Further elaborate on limitations regarding sample representativeness, generalizability, and potential biases stemming from self-reporting and selection methods	Thank-you for highlighting. We discussed these limitations in depth as a group of researchers but accept that they have not been made sufficiently explicit in the manuscript. Limitations section expanded to address representativeness (lines 402-3), generalizability (line 406) and potential biases (line 395-7).

VERSION 2 - REVIEW

**REVIEWER NAME**	Jindal, Atul
**REVIEWER AFFILIATION**	All India Institute of Medical Sciences, Pediatrics
**REVIEWER CONFLICT OF INTEREST**	No
**AI DISCLOSURE**	No, I have not used AI
**DATE REVIEW RETURNED**	23-Dec-2025

**GENERAL COMMENTS**	Can be accepted
